# CEACAM6 as detected by the AP11 antibody is a marker notable for mucin-producing adenocarcinomas

**DOI:** 10.1007/s00428-014-1688-1

**Published:** 2014-11-27

**Authors:** Ok-Jun Lee, Seung-Myoung Son, Kwon Pyo Hong, Yong-Moon Lee, Min-Young Kim, Jae-Woon Choi, Sang-Jeon Lee, Young-Jin Song, Hak Soon Kim, Wun-Jae Kim, See-Ok Shin, Hyung Geun Song

**Affiliations:** 1 Department of Pathology, Chungbuk National University College of Medicine, 52 Naesudong-ro, Heungduk-gu, Cheongju, 361-763 Korea; 2 DiNonA Incorporation, Seoul, Korea; 3 Department of Surgery, Chungbuk National University College of Medicine, Cheongju, Korea; 4 Department of Obstetrics and Gynecology, Chungbuk National University College of Medicine, Cheongju, Korea; 5 Department of Urology, College of Medicine, Chungbuk National University, Cheongju, Korea; 6 Department of Otolaryngology, College of Medicine, Chungbuk National University, Cheongju, Korea

**Keywords:** CEACMA6, AP11, Monoclonal antibody, Tumor marker

## Abstract

A new monoclonal antibody recognizing CEACAM6, which we named AP11, was generated by immunizing BALB/c mice with phytohemagglutinin-activated human peripheral blood mononuclear cells. This study aims to evaluate whether CEACAM6 can serve as a tumor marker using AP11. We examined the expression of CEACAM6 with AP11 in 11 human carcinoma cell lines by flow cytometry and 439 human tissues including 282 tumor tissues and 157 normal tissues by immunohistochemistry. CEACAM6 epitope recognized by AP11 was well preserved in formalin-fixed and paraffin-embedded tissues. Adenocarcinomas of the stomach (86 %), colorectum (95 %), pancreas (100 %), and lung (83 %), urinary bladder (100 %), and mucinous ovarian tumors (88 %) had a high rate of CEACAM6 immunoreactivity. We observed a variable expression of CEACAM6 in hepatocellular carcinomas (35 %), squamous cell carcinomas of the lung (60 %), renal cell carcinomas (14 %), urothelial carcinomas (13 %), serous carcinomas of the ovary (17 %), and breast carcinomas (11 %). Small-cell carcinomas of the lung, prostatic adenocarcinomas, papillary thyroid carcinomas, malignant melanomas, giant cell tumors, and osteosarcomas were negative for CEACAM6. All normal tissues of various organs were negative for CEACAM6. In conclusion, CEACAM6 as detected by AP11, may serve as a marker for mucin-producing adenocarcinomas of the gastrointestinal tract and ovary as well as non-small cell lung cancer. Thus, AP11 represents a valuable diagnostic tool for detecting CEACMA6-positive cancers.

## Introduction

The carcinoembryonic antigen (CEA) gene family belongs to the immunoglobulin superfamily [1] and comprises of 29 genes that are located closely together on the long arm of chromosome 19 [2]. CEA (CD66e and CEACAM5) is a complex, highly glycosylated macromolecule. It was first described in 1965 as a gastrointestinal oncofetal protein that is expressed during fetal life but not in healthy adults, re-emerging in cancer [3]. It is now accepted that this original description does not apply to CEA. Although CEA is overexpressed in a majority of carcinomas, including those of the gastrointestinal, respiratory, and genitourinary tracts as well as in breast cancers, it is also expressed in normal adult tissue [4]. Among the main proteins of the CEA family, CEA is the only one that has been characterized as a useful tumor marker in cancer patients. Increased CEA levels are the first indicator of recurrent disease [5, 6], and serum CEA levels are also used as a prognostic indicator in colorectal cancer patients.

CEACAM6 (CD66c, NCA-90) is a member of the CEA family. It is expressed on granulocytes and on the epithelium of various organs [7]. CEACAM6 is also expressed in many human cancers and is observed in the sera of cancer patients [4, 8–10]. Although CEACAM6 belongs to CEA family, the levels of CEACAM6 do not correlate with the expression of CEA [10]. CEACAM6 expression in colorectal cancer inversely correlates with cellular differentiation [11] and is an independent prognostic factor associated with a higher risk of colorectal cancer relapse [12]. CEACAM6 exhibits homotypic binding with other members of the CEA family and heterotypic interactions with integrin receptors [13]. CEACAM6 has been shown to play a role in cell adhesion, invasion, and metastasis. Antibodies that target the N-domain of CEACAM6 interfere with cell-cell interactions [14]. The apoptotic response in normal cells to inadequate or inappropriate intercellular or matrix attachment is termed anoikis [15]. While anoikis is thought to maintain tissue order within multisystem organisms by preventing ectopic cellular proliferation, resistance to anoikis is a characteristic of tumor cells and has been reported to contribute to primary tumorigenesis and metastasis in a number of cancers [16–18]. CEACAM6 overexpression markedly inhibits anoikis [19]. CEACAM6 gene silencing by small interfering RNA increases susceptibility to caspase-mediated anoikis, decreases Akt phosphorylation under anchorage-independent growth conditions, and inhibits metastatic potential [20]. Overexpression of CEACAM6 is also associated with enhanced Src-dependent cellular invasion [21].

A variety of tumor markers is widely used to detect early cancers and recurrence or metastasis after curative management for many types of malignancies. The metastatic process consists of a series of sequential steps [22]. Eradication of metastatic disease is critical for achieving survival in most cancer patients. A relatively high rate of CEACAM6-positive reactivity has been demonstrated in the sera of patients with lung cancer, hepatocellular carcinoma, pancreatic cancer, breast cancer, and colorectal cancer [10]. It has also been demonstrated that the expression levels of CEACAM6 are higher than those of CEA in malignant tumors including breast cancer, lung cancer, prostate cancer, colon cancer, pancreatic cancer, and ovarian cancer. Moreover, the level of CEACAM6 in liver metastases of colon cancers was higher than in many corresponding primary tumors [23]. Thus, CEACAM6 may be more effective than CEA as a marker for detecting early cancers and cancer relapse or metastasis. CEACAM6 has also been shown to be involved in the metastatic process and therefore it represents a potential therapeutic target for the control of metastasis.

We generated a new monoclonal antibody (mAb) AP11, which recognizes human CEACAM6. We used AP11 to analyze CEACAM6 expression by immunohistochemistry in 282 human tumor tissues. The goals of the current study were to (1) demonstrate the use of paraffin-embedded tissue blocks and cell lines to confirm the expression pattern of AP11-reactive CEACAM6 in different types of solid tumors, and (2) develop evidence that supports the utility of CEACAM6 as a tumor marker.

## Materials and methods

### Production of monoclonal antibody

Six week-old BALB/c mice were immunized intraperitoneally with 10^7^ phytohemagglutinin (PHA)-activated human peripheral blood mononuclear cells (PBMCs) at 2-week intervals for 2 months. Spleens were removed, and 10^8^ spleen cells were fused with 10^7^ SP2/0-Ag14 mouse myeloma cells using polyethylene glycol (PEG 4000, Rathway, NL, USA). The cell hybrids were cultured in flat-bottom microculture trays at 37 °C in an atmosphere of humidified air containing 5 % CO_2_, in hypoxanthine-aminopterin-thymidine (HAT) selection media (Sigma-Aldrich, USA). After 10 days, culture supernatants were harvested and tested for reactivity to human granulocytes by indirect immunofluorescence and flow cytometry (Coulter, USA). One of the resulting hybridoma clones whose supernatants were reactive to human granulocytes was named AP11. AP11 was subcloned by limiting dilution, and the culture supernatants of the sub-clones were tested for antibody production.

### Cloning of CEACAM6-hFc

Deletion mutants of CEACAM6 cDNA were derived from human lung adenocarcinoma cell line A549 by RT-PCR and ligated into pGEM-T vector (Promega, USA). The CEACAM6 cDNA was inserted into the mammalian expression vector pcDNA3.1 using restriction endonucleases *EcoR*I. The CEACAM6 cDNA fragment and human Fc cDNA were inserted into the mammalian secretion vector pSecTag (Invitrogen, USA) using *Hind*III, *EcoR*V, and *Xho*I. A day before transfection, 293T cells were cultured and maintained at 1 × 10^6^ cells/mL density on six-well plates with 3 mL DMEM medium containing 10 % heat-inactivated fetal bovine serum (FBS; GIBCO, Invitrogen, USA), incubated at 37 °C with 5 % CO_2_. The dishes were 80 % confluent on the day of transfection. Deletion mutants of CEACAM6 in pSecTag vector were transiently transfected into 293T cell by using Effectene transfection reagent kit (QIAGEN, Germany). Three days later, the resulting supernatant was tested by ELISA assay.

### ELISA method

Deletion mutants of CEACAM6 recombinant protein were tested for reactivity against AP11, goat anti-human Fc fragment, and human immunoglobulin by ELISA. Microtitration plates (96-well, Maxisorp; Nunc, Denmark) were coated with 100 ng/well of goat anti-human Fc fragment in 1× phosphate-buffered saline (PBS) and incubated with culture supernatant for 1 h at 37 °C, followed by three washes. Next, the plates were treated with the CEACAM6 recombinant protein supernatant (100 μL) and the recombinant proteins of other CEACAM families for 1 h at 37 °C, washed three times and then treated with anti CEACAM6-horseradish peroxidase (HRP) for 30 min at 37 °C. Following three washes, the plate was incubated with 50 mL/well TMB (3, 3′, 5, 5′-tetramethylbenzidine) substrate solution (Sigma-Aldrich, USA) for 10 min at room temperature. Substrate development was stopped by the addition of 2 N sulfuric acid, and the absorbance was measured at 450 nm using an ELISA reader (SpectraMax M5, Molecular devices, USA).

### Western blot analysis

Peripheral blood neutrophils from healthy donors were lysed in lysis buffer (1 % NP-40 in 50 mM Tris-HCl, pH 7.4, 50 mM EDTA, and 1 mM PMSF). After centrifugation to remove cell debris, the supernatant was subjected to 8 % SDS-PAGE under non-reducing condition with appropriate molecular weight markers. After electrophoretic transfer of the proteins to nitrocellulose membrane, the membrane was blocked with 5 % skim milk in Tris-buffered saline (10 mM Tris-HCl, 150 mM NaCl, pH 7.6) containing 0.05 % Tween-20. The membrane was incubated with AP11, followed by peroxidase-conjugated goat anti-mouse IgG (Sigma, Saint Louis, 1:5000 dilution in blocking solution). Immunoreactive proteins were visualized using the ECL chemoluminescence detection system (Amersham Pharmaciam, Sweden).

### Tissue samples and immunohistochemical staining

This study was approved by the ethics committee of Chungbuk National University. Written informed consent was obtained from the patients and patients’ families before the beginning of the study. Tissue samples from 282 human malignant tumors to 157 normal tissues were obtained from the surgical pathology files of Chungbuk National University Hospital and Korean Cancer Center as listed in Table 1. All tissues had been fixed in 10 % formalin and embedded in paraffin. Antigen retrieval was performed by microwave heating in 10 mM sodium citrate, pH 6.0 (Dako, Denmark) for 15 min. Indirect immunoperoxidase method was used.

**Table 1:** Organ type and number of tumor and normal tissues examined in this study

Organ	Tumor	Normal
Stomach	38	20
Adenocarcinoma	28	
High-grade GED	5	
Low-grade GED	5	
Colorectum	29	10
Adenocarcinoma	19	
Tubular adenoma	5	
Hyperplastic polyp	5	
Pancreatic (adenocarcinoma)	2	2
Liver (hepatocellular carcinoma)	17	10
Lung	58	30
Adenocarcinoma	18	
Squamous cell carcinoma	20	
Small cell carcinoma	20	
Ovary	16	10
Mucinous carcinoma	8	
Serous carcinoma	6	
Endometrioid carcinoma	1	
Clear cell carcinoma	1	
Kidney (renal cell carcinoma)	22	10
Bladder	33	15
Urothelial carcinoma	30	
Adenocarcinoma	3	
Prostate (adenocarcinoma)	8	8
Urethra (adenocarcinoma)	1	1
Breast (invasive ductal carcinoma)	9	9
Thyroid (papillary carcinoma)	8	8
Skin (melanoma)	4	4
Bone	37	20
Osteosarcoma	32	
Giant cell tumor	5	
Total	282	157

*N* number of samples examined

The sections were incubated overnight with primary mAb (AP11, 1:1000) in blocking buffer (4 % skim milk, 0.1 % Tween-20 in 1× PBS) at 4 °C. On the next day, the sections were washed and incubated with biotinylated goat anti-mouse IgG and HRP-conjugated streptavidin (Dako, Denmark) for 20 min at room temperature. 3,3′-Diaminobenzidine (Sigma, Saint Louis, USA) was used as chromogen. The pattern of reactivity was analyzed based on serial hematoxylin-eosin-stained sections. CEACAM6 expression detected by AP11 was defined as positive when staining was observed along the luminal cell border or in the cytoplasm in ≥10 % of cells in the whole tissue section.

## Results

### Characterization of the AP11 target antigen

**Fig. 1:**
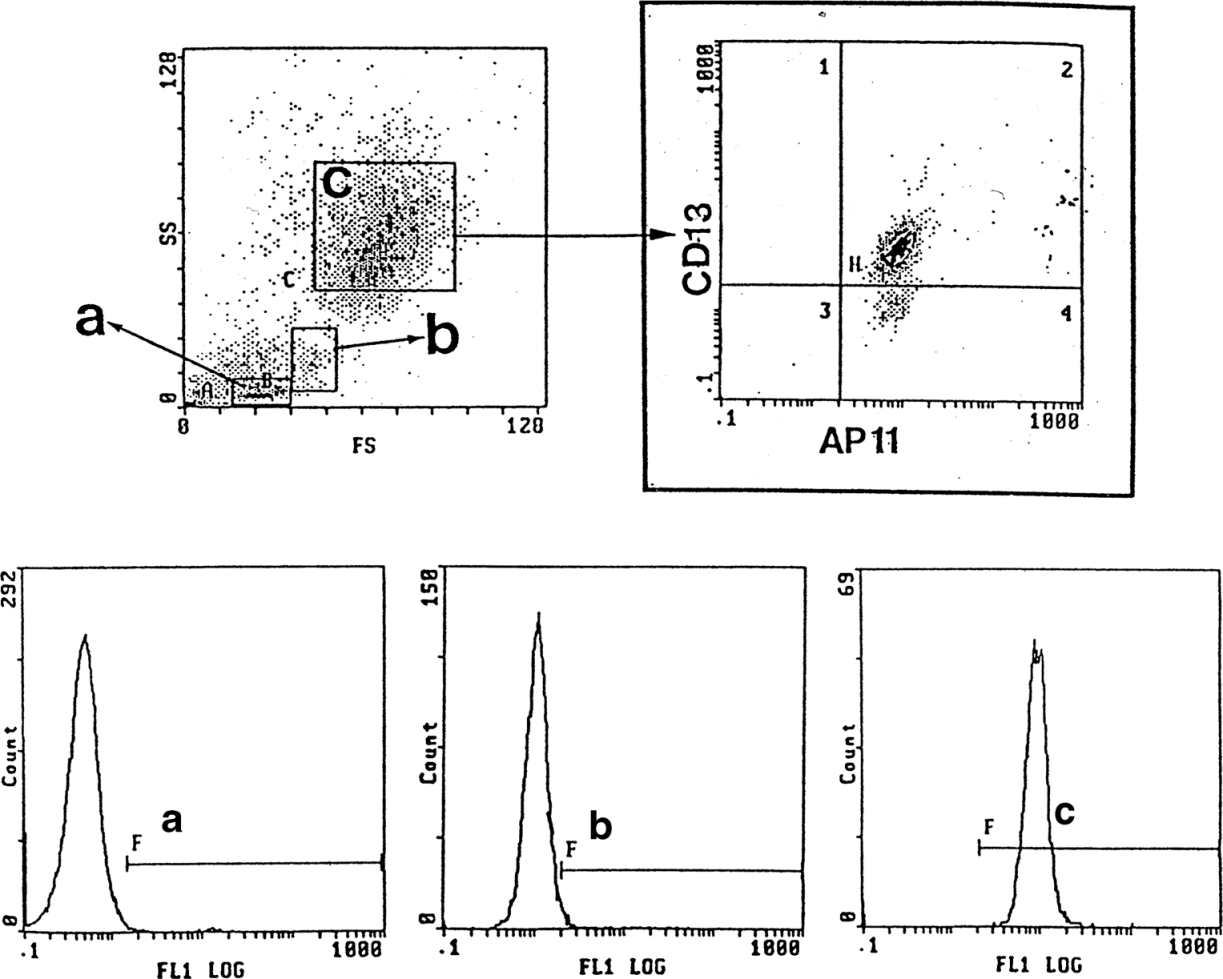
In-flow cytometric analysis of peripheral blood using mAb AP11. Granulocytes show a high level of reactivity to mAb AP11. Granulocytes (*c*) were gated to determine the CD13 and AP11 positivity (*upper right panel*). *Lower panels* show fluorescence for AP11 for lymphocytes (*a*), monocytes (*b*), and granulocytes, respectively

**Fig. 2:**
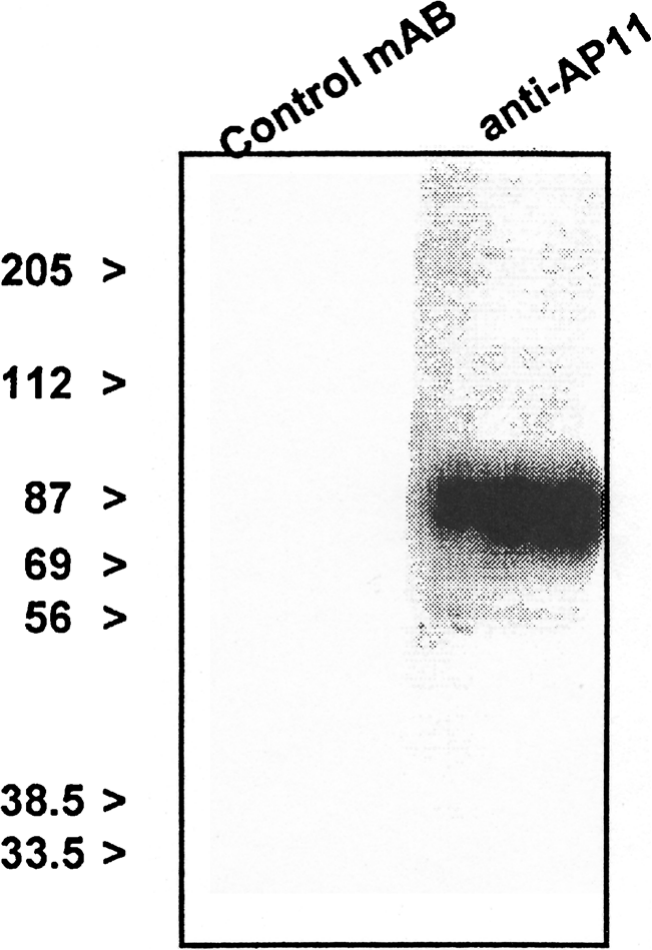
Western blot analysis demonstrating that AP11 antigen detects a protein of approximately 90 kDa in neutrophil lysates

**Fig. 3:**
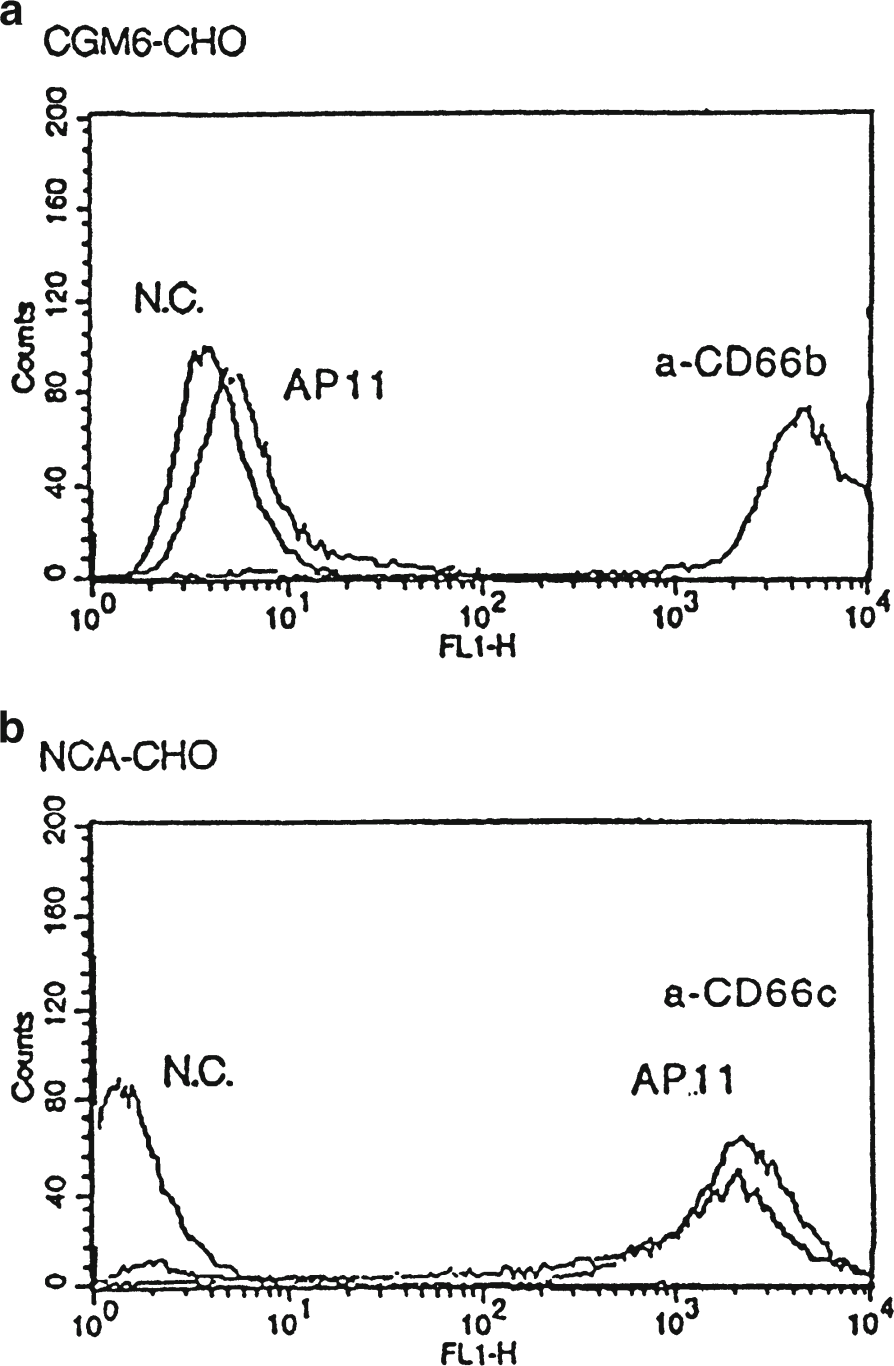
Flow cytometry result showing that AP11 is specific for CEACAM6 and does not cross-react with CD66b in a CHO transfection study. **a** CD66b transfection. **b** CEACAM6 transfection. (*N.C.* negative control)

When the peripheral blood cells were analyzed by flow cytometry using AP11, granulocytes exhibited a high level of reactivity to AP11, indicating the expression of AP11 antigen, whereas lymphocytes and monocytes were negative for AP11 (Fig. 1). Using Western blot with neutrophil lysates, the molecular weight of the AP11 antigen was shown to be approximately 90 kDa (Fig. 2). Enzyme-based immunoassay determined the isotype of AP11 to be IgM. In a separate experiment involving flow cytometric analysis, AP11 was confirmed to be highly specific to CEACAM6 (CD66c) not to cross-react with CD66b (Fig. 3).

### Epitope mapping of mAb AP11

**Fig. 4:**
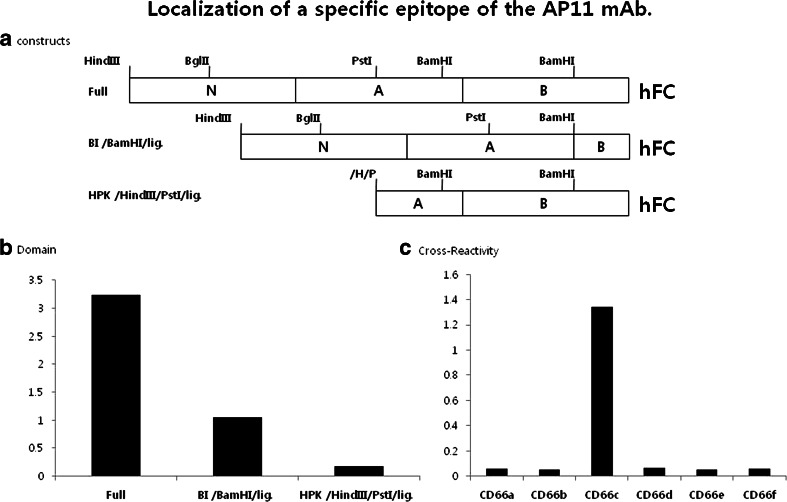
**a** Schematic diagram of recombinant CEACAM6-hFC fusion genes. The constructs for CEACAM6-hFc fusion proteins deleted in the extracellular domain were cloned to identify the AP11 epitope. **b** Sandwich ELISA performed using goat anti-hFc specific antibody and AP11 for the detection of various CEACAM6-hFc fusion proteins. The results identified N-domain of CEACAM6 as the AP11-reactive epitope. **c** ELISA results showing that AP11 only recognizes CEACAM6 among families of hFc fusion proteins, when checked for cross-reactivity using AP11 (*y*-axis, optical density)

Human CEACAM6 consists of an extracellular domain (ECD) containing one N-terminal V-type Ig-like domain and two C2-type Ig-like domains, and a hydrophobic C-terminal propeptide. The GPI membrane anchor is attached at the C-terminus following cleavage of the propeptide [24]. To identify the epitope for human CEACAM6 recombinant protein that binds to AP11, epitope mapping was performed using human Fc fusion protein gene constructs encoding CEACAM6 deletion mutants (Fig. 4a). Sandwich ELISA results showed that AP11 successfully recognized full-length protein and BI/*BamH*I/lig protein, whereas HKP/*Hind*III/*pst*I/lig protein was not recognized by AP11 (Fig. 4b). These results revealed that the AP11 epitope is located in the functional N-domain of CEACAM6. When ELISA was performed to analyze the cross-reactivity of AP11 with members of the CEACAM family, AP11 was found to be reactive only with CEACAM6 fusion protein, indicating specificity of AP11 for CEACAM6 (Fig. 4c).

### CEACAM6 expression in various tumor and normal tissues

**Fig. 5:**
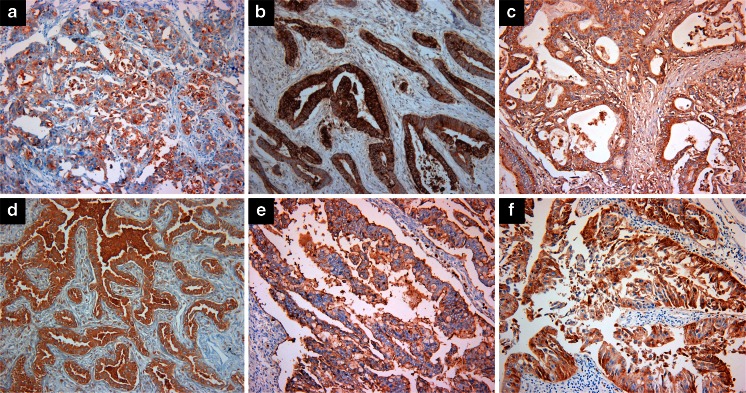
CEACAM6 immunoreactivity in **a** gastric adenocarcinoma, **b** colorectal adenocarcinoma, **c** pancreatic adenocarcinoma, **d** lung adenocarcinoma, **e** ovarian mucinous carcinoma, and **f** urinary bladder adenocarcinoma. (Magnification, ×200)

Of the gastric adenocarcinomas, 24 of 28 (86 %) showed CEACAM6 expression (Fig. 5a). In contrast, none of the cases of dysplasia including both high- and low-grade lesions expressed CEACAM6. These results suggest that AP11 differentiates between gastric cancer and its premalignant precursors.

Most colorectal adenocarcinomas (18 out of 19 cases) were positive for CEACAM6 (Fig. 5b). Four of five tubular adenomas (80 %) and four of five hyperplastic polyps (80 %) stained positive for CEACAM6. In contrast to the stomach, most premalignant lesions were also positive. Both cases of pancreatic adenocarcinoma expressed CEACAM6 (Fig. 5c), whereas only 6 out of 17 hepatocellular carcinoma samples were CEACAM6 positive.

Overall, 47 % of the lung cancer samples showed CEACAM6 expression. CEACAM6 was not expressed in small cell lung cancer (SCLC) but in 71 % of non-small cell lung cancer (NSCLC). Of the NSCLCs, lung adenocarcinomas more often showed CEACAM6 expression than squamous cell carcinomas (Fig. 5d). This expression pattern suggests that AP11 can effectively distinguish between NSCLCs and SCLCs.

In ovarian cancers, CEACAM6 expression was highest in mucinous carcinoma (seven out of eight samples, 88 %) (Fig. 5e), whereas only 17 % of serous carcinoma samples were positive for CEACAM6. None of the endometrioid and clear cell cancers exhibited CEACAM6 expression.

Adenocarcinomas from the urinary bladder (Fig. 5f) and urethra were CEACAM6 positive, whereas all prostate adenocarcinomas were CEACAM6 negative. CEACAM6 was expressed in a subset of clear renal cell carcinomas and urothelial carcinomas (14 and 13 %, respectively).

**Fig. 6:**
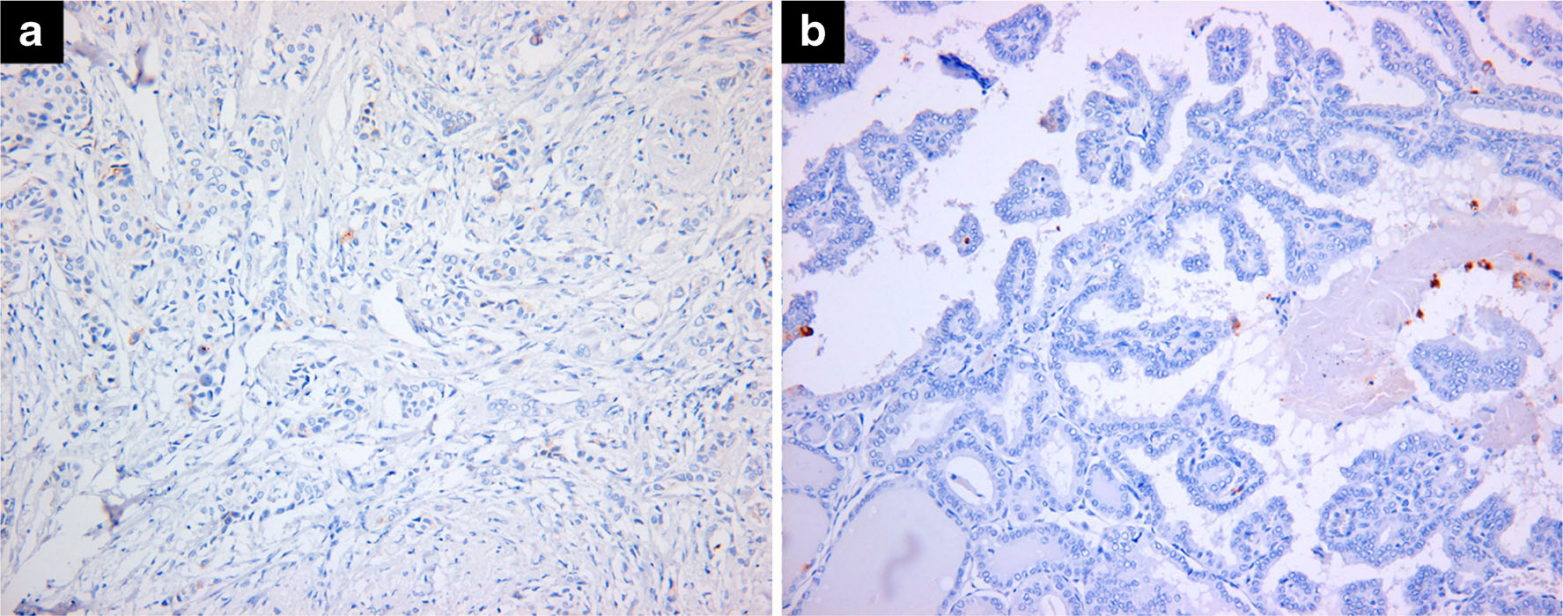
CEACAM6 is not expressed in **a** invasive ductal carcinoma of breast and **b** papillary thyroid carcinoma. (Magnification, ×200)

Only one out of nine ductal carcinomas of the breast showed CEACAM6 expression (Fig. 6a), whereas no papillary thyroid carcinoma displayed CEACAM6 expression (Fig. 6b). Of the 4 melanomas, 32 osteosarcomas, and 5 giant cell tumors of the bone, none expressed CEACAM6 (Table 2).

**Table 2:** Profile of CEACAM6-positive immunostaining in various tumor tissues

Organ	Diagnosis	*N* (282)	Positive *N* (%)
Stomach	Adenocarcinoma	28	24 (86)
	High-grade GED	5	0 (0)
	Low-grade GED	5	0 (0)
Colorectum	Adenocarcinoma	19	18 (95)
	Tubular adenoma	5	4 (80)
	Hyperplastic polyp	5	4 (80)
Pancreas	Adenocarcinoma	2	2 (100)
Liver	Hepatocellular carcinoma	17	6 (35)
Lung	Adenocarcinoma	18	15 (83)
	Squamous cell carcinoma	20	12 (60)
	Small cell carcinoma	20	0 (0)
Ovary	Mucinous carcinoma	8	7 (88)
	Serous carcinoma	6	1 (17)
	Endometrioid carcinoma	1	0 (0)
	Clear cell carcinoma	1	0 (0)
Kidney	Renal cell carcinoma	22	3 (14)
Bladder	Urothelial carcinoma	30	4 (13)
	Adenocarcinoma	3	3 (100)
Prostate	Adenocarcinoma	8	0 (0)
Urethra	Adenocarcinoma	1	1 (100)
Breast	Invasive ductal carcinoma	9	1 (11)
Thyroid	Papillary carcinoma	8	0 (0)
Skin	Melanoma	4	0 (0)
Bone	Osteosarcoma	32	0 (0)
Bone	Giant cell tumor	5	0 (0)

*GED* gastric epithelial dysplasia, *N* number of samples examined

None of normal tissue samples from the stomach, colorectum, pancreas, liver, lung, breast, thyroid, ovary, kidney, bladder, prostate, urethra, skin, and bone showed CEACAM6 immunoreactivity.

## Discussion

CEACAM6 (CD66c, NCA-90) is a 90-kDa glycosylphosphatidylinositol (GPI)-linked glycoprotein that is overexpressed in a variety of gastrointestinal malignancies [12, 25]. Despite lacking a transmembrane or intracellular domain, CEACAM6 influences intracellular signal transduction and plays an important role in the progression of gastrointestinal cancer [8, 11, 25]. To date, pancreatic and colorectal cancers have been the primary focus of CEACAM6 expression studies [8, 9, 12, 18–20, 25–28]. We show that CEACAM6 is expressed in particular in mucin-producing, gland-forming adenocarcinomas that arise in the gastrointestinal tract, lung, and ovary. Blumenthal et al. explored the expression of CEACAM6 and CEA in a panel of solid tumors, including breast, lung, ovary, prostate, pancreas, and colorectal cancers, using tissue microarrays [23]. Their results show that CEACAM6 is more strongly expressed than CEA, and that expression of CEACAM6 is strongly dependent on the histologic tumor type. CEACAM6 expression in some subtypes was 2–4-fold stronger than in normal tissue, while in others, expression was similar to that in non-neoplastic tissues. Adenocarcinomas of the lung expressed CEACAM6 more strongly than squamous cell carcinomas. CEACAM6 expression in large cell and poorly differentiated squamous carcinomas was similar to non-neoplastic lung tissue, and SCLC also expressed CEACAM6. We did not detect CEACAM6 expression in SCLC, while adenocarcinomas and squamous cell carcinomas frequently expressed CEACAM6 (83 and 60 %, respectively). These findings point indicate that AP11 stained CEACAM6 is a marker that differentiates between SCLC and NSCLC in tissue samples.

CEACAM6 is highly expressed in ovarian mucinous neoplasms. Litkouhi et al. reported that CEACAM6 is expressed at higher levels in borderline mucinous neoplasms and invasive mucinous carcinomas than in serous ovarian neoplasms [29]. Consistent with this, we found that seven out of eight mucinous carcinomas (88 %) but only one case of serous carcinoma (17 %) stained positive for CEACAM6 using the AP11 antibody. Taken together, these data suggest that CEACMA6 may be a potential marker for mucinous ovarian carcinomas.

Kinugasa et al. investigated CEACAM6 expression in gastric adenocarcinoma and carcinoma cell lines in comparison to adjacent normal gastric mucosa using reverse transcription-polymerase chain reaction (RT-PCR). They found higher CEACAM6 transcript levels in gastric cancer tissues compared to adjacent normal tissues, and CEACAM6 transcripts were detectable in 92 % (11/12) and 25 % (3/12) of gastric cancer and normal gastric mucosa samples, respectively [30]. Yasui et al. demonstrated that CEACAM6 expression is upregulated in gastric cancer compared to normal gastric epithelia using serial analysis of gene expression (SAGE) [31]. Kodera et al. showed similar upregulation of CEA and CEACAM6 in gastric carcinomas using Northern blot analysis [25]. We observed differential expression of the AP11-reactive CEACAM6 epitope in invasive gastric cancer and dysplastic lesions. We observed high expression (86 %) in gastric cancer, which is consistent with previous reports, but no or low CEACAM6 expression in dysplasia. Taken together, these findings suggest that CEACAM6 is a potentially useful marker for gastric cancer and differentiates between carcinomas and gastric dysplasia.

Upregulation of CEACAM6 expression in hyperplastic polyps and early adenomas indicates that it is an early event in the development of colorectal cancer. Schozel et al. found CEACAM6 expression in all 25 examined colonic polyps [8]. We observed expression of CEACMA6 in most tubular adenomas (80 %) and hyperplastic polyps (80 %). A prognostic significance of CEA expression for different types of carcinoma has not been established [32–35]. In contrast, expression of CEACAM6 in colorectal cancer tissue is significantly associated with poor prognosis [12]. Tissue expression of CEACAM6 in liver metastases of colorectal cancer is higher than that in primary colorectal cancer [23]. However, in breast, colon, or lung carcinomas, CEACAM6 expression is similar to that in their lymph node metastases [23]. CEACAM6 plays an important role in migration, invasion, and adhesion, crucial steps for the metastatic spread of cancer cells to secondary tissue sites other than lymph nodes and potential targets for new therapeutic modalities [36–38].

Pancreatic cancer has been the most extensively studied neoplasm with respect to CEACAM6 expression [19–21, 23, 26–28]. The majority of invasive pancreatic adenocarcinomas express CEACAM6, and CEACAM6-negative patients may represent a subgroup of patients who survive longer after surgical resection [28]. The level of CEACAM6 expression influences the cellular invasiveness of pancreatic adenocarcinomas in a c-Src-dependent manner. Overexpression of CEACAM6 enhances cellular invasion, whereas CEACAM6 knockdown attenuates invasiveness [21]. CEACAM6 is an important determinant of malignant cellular behavior in pancreatic adenocarcinoma. Anoikis refers to the apoptotic response induced in normal cells by inadequate or inappropriate adhesion to a substrate [15]. It is postulated that resistance to anoikis contributes to primary tumorigenesis and metastasis [16–18]. Anoikis resistance is associated with increased CEACAM6 expression, and CEACAM6 gene silencing impairs anoikis resistance and inhibits the metastatic potential of pancreatic adenocarcinoma cells in vivo [19]. CEACAM6 expression appears to be an early event in the progression to pancreatic cancer. In a previous study, half of the low-grade pancreatic intraepithelial neoplasia (PanIN) lesions examined demonstrated some positive staining for CEACAM6, whereas all PanIN3 lesions were CEACAM6 positive [28].

The expression of CEACAM6 in atypical ductal hyperplasia of the breast (ADH) is strongly associated with the development of invasive breast cancer and might serve as a marker for its early diagnosis. It also represents a potential molecular target for the treatment of patients with precancerous lesions who express CEACAM6 to prevent the development of invasive breast cancer [39]. In recent study, CEACAM6 was reported as predictive for endocrine therapy resistance and a unique mediator of migration and invasion of drug-resistant estrogen-deprived breast cancer cells [40, 41].

The level of CEACAM6 in the blood of normal individuals is higher (0.05 mg/L) than that of CEA (<2 μg/L). It is therefore unlikely that CEACAM6 will be more useful than CEA as a serum marker [4]. The expression of CEACAM6 in solid tumors is higher than that of CEA, which makes it a more promising target for antibody-based anti-metastatic and chemosensitizing therapy [23].

In summary, we generated a new mAb, AP11, which recognizes CEACAM6, and evaluated its ability to be used as a tool to recognize CEACAM6 expression as a tumor marker in human cancer cells and tissues. AP11 recognizes CEACAM6 in formalin-fixed and paraffin-embedded tissues. CEACAM6 expression is high in particular in mucin-producing, gland-forming adenocarcinomas arising in the gastrointestinal tract, ovary, and NSCLCs. Thus, mAb AP11-stained CEACAM6 may serve as a marker for mucinous tumors.

## References

[1] Thompson JA (1995). Molecular cloning and expression of carcinoembryonic antigen gene family members. Tumour Biol.

[2] Zimmermann W, Weber B, Ortlieb B, Rudert F, Schempp W, Fiebig HH, Shively JE, von Kleist S, Thompson JA (1988). Chromosomal localization of the carcinoembryonic antigen gene family and differential expression in various tumors. Cancer Res.

[3] Gold P, Freedman SO (1965). Specific carcinoembryonic antigens of the human digestive system. J Exp Med.

[4] Hammarstrom S (1999). The carcinoembryonic antigen (CEA) family: structures, suggested functions and expression in normal and malignant tissues. Semin Cancer Biol.

[5] Minton JP, Hoehn JL, Gerber DM, Horsley JS, Connolly DP, Salwan F, Fletcher WS, Cruz AB, Gatchell FG, Oviedo M (1985). Results of a 400-patient carcinoembryonic antigen second-look colorectal cancer study. Cancer.

[6] Wanebo HJ, Llaneras M, Martin T, Kaiser D (1989). Prospective monitoring trial for carcinoma of colon and rectum after surgical resection. Surg Gynecol Obstet.

[7] Kuroki M, Matsuo Y, Kinugasa T, Matsuoka Y (1992). Three different NCA species, CGM6/CD67, NCA-95, and NCA-90, are comprised in the major 90 to 100-kDa band of granulocyte NCA detectable upon SDS-polyacrylamide gel electrophoresis. Biochem Biophys Res Commun.

[8] Scholzel S, Zimmermann W, Schwarzkopf G, Grunert F, Rogaczewski B, Thompson J (2000). Carcinoembryonic antigen family members CEACAM6 and CEACAM7 are differentially expressed in normal tissues and oppositely deregulated in hyperplastic colorectal polyps and early adenomas. Am J Pathol.

[9] Hinoda Y, Saito T, Takahashi H, Itoh F, Adachi M, Imai K (1997). Induction of nonspecific cross-reacting antigen mRNA by interferon-gamma and anti-fibronectin receptor antibody in colon cancer cells. J Gastroenterol.

[10] Kuroki M, Matsushita H, Matsumoto H, Hirose Y, Senba T, Yamamoto T (1999). Nonspecific cross-reacting antigen-50/90 (NCA-50/90) as a new tumor marker. Anticancer Res.

[11] Ilantzis C, DeMarte L, Screaton RA, Stanners CP (2002). Deregulated expression of the human tumor marker CEA and CEA family member CEACAM6 disrupts tissue architecture and blocks colonocyte differentiation. Neoplasia.

[12] Jantscheff P, Terracciano L, Lowy A, Glatz-Krieger K, Grunert F, Micheel B, Brummer J, Laffer U, Metzger U, Herrmann R, Rochlitz C (2003). Expression of CEACAM6 in resectable colorectal cancer: a factor of independent prognostic significance. J Clin Oncol.

[13] Stanners CP, Fuks A (1998). Properties of adhesion mediated by the human CEA family. Cell adhesion and communication by the CEA family.

[14] Yamanka T, Kuroki M, Matsuo Y, Matsuoka Y (1996). Analysis of heterophilic cell adhesion mediated by CD66b and CD66c using their soluble recombinant proteins. Biochem Biophys Res Commun.

[15] Frisch SM, Francis H (1994). Disruption of epithelial cell-matrix interactions induces apoptosis. J Cell Biol.

[16] Yawata A, Adachi M, Okuda H, Naishiro Y, Takamura T, Hareyama M, Takayama S, Reed JC, Imai K (1998). Prolonged cell survival enhances peritoneal dissemination of gastric cancer cells. Oncogene.

[17] Streuli CH, Gilmore AP (1999). Adhesion-mediated signaling in the regulation of mammary epithelial cell survival. J Mammary Gland Biol Neoplasia.

[18] Shanmugathasan M, Jothy S (2000). Apoptosis, anoikis and their relevance to the pathobiology of colon cancer. Pathol Int.

[19] Duxbury MS, Ito H, Zinner MJ, Ashley SW, Whang EE (2004). CEACAM6 gene silencing impairs anoikis resistance and in vivo metastatic ability of pancreatic adenocarcinoma cells. Oncogene.

[20] Duxbury MS, Ito H, Benoit E, Waseem T, Ashley SW, Whang EE (2004). A novel role for carcinoembryonic antigen-related cell adhesion molecule 6 as a determinant of gemcitabine chemoresistance in pancreatic adenocarcinoma cells. Cancer Res.

[21] Duxbury MS, Ito H, Benoit E, Ashley SW, Whang EE (2004). CEACAM6 is a determinant of pancreatic adenocarcinoma cellular invasiveness. Br J Cancer.

[22] Gupta GP, Massague J (2006). Cancer metastasis: building a framework. Cell.

[23] Blumenthal RD, Leon E, Hansen HJ, Goldenberg DM (2007). Expression patterns of CEACAM5 and CEACAM6 in primary and metastatic cancers. BMC Cancer.

[24] Tawaragi Y, Oikawa S, Matsuoka Y, Kosaki G, Nakazato H (1988). Primary structure of nonspecific crossreacting antigen (NCA), a member of carcinoembryonic antigen (CEA) gene family, deduced from cDNA sequence. Biochem Biophys Res Commun.

[25] Kodera Y, Isobe K, Yamauchi M, Satta T, Hasegawa T, Oikawa S, Kondoh K, Akiyama S, Itoh K, Nakashima I (1993). Expression of carcinoembryonic antigen (CEA) and nonspecific crossreacting antigen (NCA) in gastrointestinal cancer; the correlation with degree of differentiation. Br J Cancer.

[26] Riley CJ, Engelhardt KP, Saldanha JW, Qi W, Cooke LS, Zhu Y, Narayan ST, Shakalya K, Croce KD, Georgiev IG, Nagle RB, Garewal H, Von Hoff DD, Mahadevan D (2009). Design and activity of a murine and humanized anti-CEACAM6 single-chain variable fragment in the treatment of pancreatic cancer. Cancer Res.

[27] Niu G, Murad YM, Gao H, Hu S, Guo N, Jacobson O, Nguyen TD, Zhang J, Chen X (2012). Molecular targeting of CEACAM6 using antibody probes of different sizes. J Control Release.

[28] Duxbury MS, Matros E, Clancy T, Bailey G, Doff M, Zinner MJ, Ashley SW, Maitra A, Redston M, Whang EE (2005). CEACAM6 is a novel biomarker in pancreatic adenocarcinoma and PanIN lesions. Ann Surg.

[29] Litkouhi B, Litkouhi B, Fleming E, Welch WR, Berkowitz RS, Birrer MJ, Mok SC (2008). Overexpression of CEACAM6 in borderline and invasive mucinous ovarian neoplasms. Gynecol Oncol.

[30] Kinugasa T, Kuroki M, Takeo H, Matsuo Y, Ohshima K, Yamashita Y, Shirakusa T, Matsuoka Y (1998). Expression of four CEA family antigens (CEA, NCA, BGP and CGM2) in normal and cancerous gastric epithelial cells: up-regulation of BGP and CGM2 in carcinomas. Int J Cancer.

[31] Yasui W, Oue N, Ito R, Kuraoka K, Nakayama H (2004). Search for new biomarkers of gastric cancer through serial analysis of gene expression and its clinical implications. Cancer Sci.

[32] Cosimelli M, De Peppo F, Castelli M, Giannarelli D, Schinaia G, Castaldo P, Buttini GL, Sciarretta F, Bigotti G, Di Filippo F (1989). Multivariate analysis of a tissue CEA, TPA, and CA 19.9 quantitative study in colorectal cancer patients. A preliminary finding. Dis Colon Rectum.

[33] Lorenzi M, Vindigni C, Minacci C, Tripodi SA, Iroatulam A, Petrioli R, Francini G (1997). Histopathological and prognostic evaluation of immunohistochemical findings in colorectal cancer. Int J Biol Markers.

[34] Cooper MJ, Mackie CR, Skinner DB, Moossa AR (1979). A reappraisal of the value of carcinoembryonic antigen in the management of patients with various neoplasms. Br J Surg.

[35] Robertson JF, Ellis IO, Bell J, Todd JH, Robins A, Elston CW, Blamey RW (1989). Carcinoembryonic antigen immunocytochemistry in primary breast cancer. Cancer.

[36] Fidler IJ (2002). The organ microenvironment and cancer metastasis. Differentiation.

[37] Blumenthal RD, Hansen HJ, Goldenberg DM (2005). Inhibition of adhesion, invasion, and metastasis by antibodies targeting CEACAM6 (NCA-90) and CEACAM5 (Carcinoembryonic Antigen). Cancer Res.

[38] Glinsky GV (1998). Anti-adhesion cancer therapy. Cancer Metastasis Rev.

[39] Poola I, Shokrani B, Bhatnagar R, DeWitty RL, Yue Q, Bonney G (2006). Expression of carcinoembryonic antigen cell adhesion molecule 6 oncoprotein in atypical ductal hyperplastic tissues is associated with the development of invasive breast cancer. Clin Cancer Res.

[40] Maraqa L, Cummings M, Peter MB, Shaaban AM, Horgan K, Hanby AM, Speirs V (2008). Carcinoembryonic antigen cell adhesion molecule 6 predicts breast cancer recurrence following adjuvant tamoxifen. Clin Cancer Res.

[41] Lewis-Wambi JS, Cunliffe HE, Kim HR, Willis AL, Jordan VC (2008). Overexpression of CEACAM6 promotes migration and invasion of oestrogen-deprived breast cancer cells. Eur J Cancer.

